# Composting of recovered rock wool from hydroponics for the production of soil amendment

**DOI:** 10.1007/s11356-024-33041-2

**Published:** 2024-04-04

**Authors:** Darja Istenič, Franja Prosenc, Neva Zupanc, Matejka Turel, Andrej Holobar, Radmila Milačič, Stefan Marković, Rok Mihelič

**Affiliations:** 1 Faculty of Health Sciences, University of Ljubljana, Zdravstvena pot 5, Ljubljana, Slovenia; 2 Faculty of Civil and Geodetic Engineering, University of Ljubljana, Jamova cesta 2, Ljubljana, Slovenia; 3 BioResource Systems Research Group, School of Civil Engineering, University of Leeds, Leeds, LS2 9JT UK; 4 Knauf Insulation d.o.o, Trata 32, Škofja Loka, Slovenia; 5 ECHO Instruments d.o.o, Zeče 25, Slovenske Konjice, Slovenia; 6 Department of Environmental Sciences, Jožef Stefan Institute, Jamova cesta 39, Ljubljana, Slovenia; 7 Department of Agronomy, Biotechnical Faculty, University of Ljubljana, Jamnikarjeva 101, Ljubljana, Slovenia

**Keywords:** Recycling, Circular economy, Horticulture, Bioassay, Hexavalent chromium, Respirometer

## Abstract

Due to its fibrous structure and high water holding capacity, rock mineral wool (RMW) has boosted the development of hydroponics. Consequently, the amount of waste RMW has also increased tremendously, which has stimulated the research and development of RMW reuse options. In this study, composting and degradability of RMW from hydroponics (gRMW) were tested in combination with different ratios of biowaste compost, including physical and chemical properties of the starting and final materials, and potential ecological hazards of the final product. gRMW had high water holding capacity and low organic matter content, which was easily degradable. Limits of toxic elements according to EU regulation were not exceeded. Degraded gRMW mixtures with compost did not exhibit toxicity to plants or aquatic bacteria and showed intermediate or limited habitat function for earthworms, which preferred the sole gRMW not mixed with compost. Overall, degraded gRMW exhibited parameters of safe soil amendment.

**Supplementary Information:**

The online version contains supplementary material available at 10.1007/s11356-024-33041-2.

## Introduction

The growing world population, problems arising from the climate change, and the loss of arable land have led to the search for new ways of growing food. Amongst them, hydroponic food production is a promising alternative to soil-based production. In addition, hydroponics can be environmentally sound, but only if it is practiced in a sustainable manner with respect to the use of fertilisers, water, and materials. The rapid development of hydroponics in the last two decades is mainly due to the use of rock mineral wool (RMW) as an artificial plant substrate. Artificial substrates in controlled environments reduce the possibility of infestation of growing crops with various diseases, pests, and weeds and allow for higher yields (Bougoul et al. 2005). RMW is a highly porous, fibrous substrate with high water holding capacity (60–80% w/w) and even distribution of water and nutrients, allowing roots to grow faster and throughout the whole volume of the substrate. It also allows adequate aeration of the roots. It is structurally stable enough to adequately support roots and stem development. The RMW used in this study is manufactured using the best available techniques to prevent, reduce, and control emissions of fibres and gases in order to comply with Note Q of EC Regulation 1272/2008. In addition, the International Agency for Research on Cancer (IARC) declared mineral wool as insufficiently proven for carcinogenicity in humans. However, the production of RMW causes carbon emissions of 1471 kg CO_2_eq/t, of which raw material production causes more than half of the emissions (Huang and Zhang 2020). To improve the environmental footprint of hydroponics, biobased substrates based on wood, hemp, flax, miscanthus, and lignite can be used (Rossouw 2015; Łaźny et al. 2022; Nguyen 2022). These materials have been shown to be more environmentally friendly than RMW (Schulte et al. 2021).

An artificial substrate can be used for several growing seasons if there are adequate porosity and aeration of the roots and if the new crop can tolerate organic exudates and soil-borne microorganisms that developed during the previous growing cycle. After a few cycles, most of the pores in the substrate are filled with old and dead roots, and poorly soluble salts from water and fertilisers accumulate, making the substrate more compact and losing its original properties (Maucieri et al. 2019). To maintain good plant growth in such systems, the used mineral wool is often replaced in each growth cycle (Łaźny et al. 2021).

Consequently, recovered RMW from hydroponics is a waste that significantly increases the environmental footprint of hydroponic food production. Recently, concerns have been raised about its disposal because it does not degrade and is a major burden on landfills (Dannehl et al. 2015). To our knowledge, there are no data on the exact amount of RMW waste from hydroponics, not even on the area of hydroponics in Europe (article search at Web of Science, Elsevier, Springer, and Google Scholar, and general search, e.g., Eurostat, Wikipedia, Google). However, assuming that the Netherlands, the largest hydroponic producer in Europe, used 55,500 t of RMW as a growing medium in 2019 and that the market for RMW is growing (Knauf Insulation internal market research), it is obvious that the amount of RMW waste is significant. According to references used by Nerlich et al. (2022), up to 150 m^3^ of substrate waste is produced per hectare of tomato cultivation per year.

In accordance with the principles of circular economy and end-of-waste criteria (i.e., when a given waste ceases to be a waste and becomes a product or a secondary raw material; Waste Framework Directive 2008/98/ EC), it is of utmost importance to investigate the possibility of reusing RMW from greenhouses (gRMW) as a raw material for other activities. There are several ways to recycle mineral wool waste, but most of the processes refer to waste generated during RMW production and to mineral wool as construction waste. The latter can be partially recycled back into the manufacturing process or processed into other products for the construction industry (Väntsi and Kärki 2014; Yap et al. 2021). Recycling of gRMW requires different approaches because it has altered physicochemical properties, is overgrown with plant roots and saturated with hydroponic growth medium salts, and thus may contain excess nutrients and trace amounts of pesticides. The exact composition of these additives varies between different hydroponic growing practices.

One of the possibilities of further use is as a structural material in the composting of organic waste. This area is still poorly understood, and the effects of the final product on the structure and fertility of the amended soil, as well as on the soil and other organisms through leaching, are unknown. In the EU, soil amendments must comply with the EU Regulation laying down rules on the making available on the market of EU fertilising products (2019/1009). This regulation defines ‘a soil improver shall be an EU fertilising product the function of which is to maintain, improve or protect the physical or chemical properties, the structure or the biological activity of the soil to which it is added’.

Some case studies are reported (Grodan 2022), particularly in North America, where a major supplier of RMW is composting over 5000 t of gRMW per year through leading composting facilities. It is recommended that composting facilities use 10–25% gRMW to 75–90% green waste (by weight). At these proportions, the gRMW decomposes quite easily and the mineral wool is no longer visible.

In this study, we fill the knowledge gap in recycling gRMW by testing the degradability of gRMW in combination with different ratios of biowaste compost. We analysed the physical and chemical properties of the starting and final materials and also evaluated potential ecological hazards of the final products from the composting process to soil and aquatic ecosystems by performing avoidance and toxicity tests on earthworms, marine bacteria, and plants. Our hypotheses were that (1) the addition of compost improves the degradability of gRMW in terms of time and degree of mineralization; (2) the physical and chemical properties of gRMW change during the composting process; (3) gRMW has no toxicity to living organisms after composting; and (4) gRMW has beneficial properties for use as a soil amendment after composting.

## Materials and methods

### Starting material

The RMW horticultural boards investigated in this study were manufactured by Knauf Insulation d.o.o., Slovenia. The concentration of the biobased organic binder in the RMW is 5 to 10% of the final product weight. The starting material for the study was recovered rock mineral wool from hydroponic cultivation of tomatoes in the Netherlands. After one growing season, the greenhouse vegetation was cut off; the gRMW was removed from the greenhouse and transported to the laboratory, where the plastic wrapping was removed; and the material with the vegetation residues was dried at 35 ± 3 °C to constant weight. The dry gRMW was ground into approximately 1 cm^2^ particles using a wood shredder and mixed with biowaste compost. The biowaste compost was produced from separately collected organic municipal waste (food waste, leaves, grass, shrub, and tree cuttings) and composted in a commercial composting plant (Kogal d.o.o., Šentilj, Slovenia). The compost was biologically well stabilised and mineralized and contained only 16% organic carbon and 1.77% total nitrogen (C_org_/N = 9). The compost was also sanitised and therefore free of pathogens (*Salmonella* sp., *Escherichia coli*). The compost batch used in this study was classified as 1st class compost according to the Slovenian regulation (decree on the treatment of biodegradable waste and the use of compost or digestate, OG RS, 2013). The detailed analysis of compost characteristics in presented in Table 1 (100% C). Before mixing with gRMW, the compost was dried and sieved through a 5-mm sieve. The following mixtures of gRMW and compost were prepared in different weight ratios: 100% compost (100% C), 10% gRMW, 50% gRMW, 90% gRMW, and 100% gRMW. Additional information on the input material can be found in the Supplementary Information.

### Degradation test

The degradation test on the mixtures of gRMW and compost was performed in the respirometer ER12 (ECHO Instruments d.o.o., Slovenia) according to modified ISO 14855–1:2012, which applies to aerobic degradation of samples and thus simulates composting. The ER12 respirometer comprised of 12 reactor vessels in a thermostatic chamber set at 58 °C in accordance with the ISO 14855 standard. CO_2_ concentration was measured with an infrared sensor, O_2_ concentration with an electrochemical sensor, temperature with a negative temperature coefficient sensor, pressure with a semiconductor, air flow with a thermal mass flow metre, and humidity with a capacitive humidity sensor. The sensors were placed in the control unit and the data was automatically fed into the ECHO software for storage and data analysis. Measurements in each channel were taken continuously for 94 days at 2.18-h intervals. The percentage of biodegradability of organic matter in the samples was calculated as per Eq. (1).

1 $${\text{Biodegradability}}\;\left(\%\right)=\frac{m\left({{\text{CO}}}_{2}\right) \times\;M\left({\text{C}}\right)}{M\left({{\text{CO}}}_{2}\right)\times\;m\left({\text{C}}\right)}\times\;100$$

where *m*(CO_2_) is the total mass of CO_2_ produced by the sample (g), *M*(C) is the molar mass of carbon (g/mol), *M*(CO_2_) is the molar mass of CO_2_ (g/mol), and *m*(C) is the initial mass of organic carbon in the sample. Additional information on the respirometer ER12 and the degradation test can be found in the Supplementary Information.

### Physical and chemical characterisation

To determine the content of volatile matter, the mass loss of the compost and gRMW mixtures was determined by subtracting the mass after combustion at 550 °C (inorganic matter) from the original mass of the samples.

The water holding capacity (WHC) of gRMW mixtures was determined by soaking the sample with water in a container for 24 h. After soaking, the excess water was allowed to drain through Whatman #2 filter paper. After draining the gravitational water, the sample was weighed. WHC was calculated as the amount of water retained by the sample based on the mass of the sample dried to constant weight at 105 °C (g water/g dry sample) or based on the volume of the dry sample (g water/L dry sample). The volume (bulk density) was measured by filling dry material into a cylinder with a sealed bottom that was exactly 1 L in size. The cylinder was dropped three times from a height of 10 cm onto the flat surface and each time filled to the top by carefully levelling the surface. After weighing the cylinder with the sample, the weight of 1 L of dry substrate (g) was obtained by subtracting the weight of the empty cylinder (Gruda and Schnitzler 1999).

pH, electrical conductivity (EC), and total carbon (C-total) and total nitrogen (N-total) were measured according to ISO 10390:2005, ISO 11265:1996, and ISO 10694:1996, respectively. The analysis for the determination of iron (Fe), aluminium (Al), molybdenum (Mo), copper (Cu), lead (Pb), zinc (Zn), nickel (Ni), manganese (Mn), arsenic (As), cadmium (Cd), calcium (Ca), phosphorous (P), chromium (Cr), magnesium (Mg), natrium (Na), potassium (K), mercury (Hg), sulphur (S), and selenium (Se) was performed following the BS EN 13650:2001 standard. The details of the analyses are described in the Supplementary Information.

Nitrate and ammonium nitrogen (NO_3_-N and NH_4_-N) content was measured in the extract prepared by adding 0.01 M CaCl_2_ (1:10 w/V) to the samples, shaking for 2 h, then centrifuging, and filtering through a 0.45-µm filter. Measurements were performed according to ISO 14255:1998 using the automatic Gallery™ instrument (Thermo Scientific™, USA).

Plant-available phosphorous (AL-P_2_O_5_) and potassium (AL-K_2_O) were determined with an ammonium lactate method (AL-method, Egnér et al. 1960). Five grams of air-dried gRMW mixtures were weighed, soaked in 100 mL of ammonium lactate solution (pH = 3.75), sealed, and shaken for 2 h at room temperature. The extract was then filtered, and P was determined spectrophotometrically with the molybdate method, while K was determined by flame atomic absorption spectrometry (FAAS) and by spectrophotometry.

Total Cr(VI) was determined in 100% gRWM before the degradation test and in degraded 50%, 90%, and 100% gRMW samples. Extraction was performed according to ISO 15192:2010 and is described in detail in the Supplementary Information. Cr(VI) content was analysed immediately with high-performance liquid chromatography inductively coupled plasma mass spectrometry (HPLC-ICP-MS). The same analytical procedure was applied to a blank sample (alkaline extracting solution). To control for species interconversion during the extraction procedure, alkaline extract containing 100% gRWM sample before the degradation was doubly spiked with ^53^Cr(III)- and ^50^Cr(VI)-enriched Cr stable isotopic solutions (Novotnik et al. 2012). To minimise procedural blanks, all chemicals used were of supra pure quality. Separation was performed on a strong anion-exchange FPLC column of Mono Q HR 5/5 (GE Healthcare Bio-Sciences, Sweden), using Agilent series 1200 quaternary pump (Japan). Separated Cr species were detected by ICP-MS on an Agilent 7900 instrument at *m/*z 52, 53, and 50 using the procedure described by Drinčić et al. (2018). To obtain a lower limit of detection (LOD), Cr(VI) content was determined by HPLC-ICP-MS, a considerably more sensitive analytical procedure compared to spectrophotometric detection, recommended in ISO 15192:2010. However, to evaluate the performance and suitability of the two methods, both were used for the analysis of gRMW alkaline extracts.

### Bioassays to evaluate the ecological hazard

#### Avoidance test with earthworms

The test was adapted from ISO 17512–1:2008 and performed on substrates that consisted of degraded 100% C, 10%, 50%, 90%, and 100% gRMW mixed with Rastko compost (JP VOKA SNAGA d.o.o., Slovenia) at a ratio of 1:3. The control substrate was 100% Rastko. Moisture was adjusted to 60% WHC. Earthworms *Lumbricus rubellus* were used, which were obtained from Biobrazda d.o.o., Slovenia.

The test was performed in plastic buckets with perforated lids. The buckets were divided into two equal parts, adding 200 g of test substrate to one half and 200 g of control substrate to the other half. Sexually mature earthworms were cleaned with tap water and placed in the control substrate for 24-h adaptation. After, the earthworms were washed again and eight earthworms were placed at the boundary between the two substrates in each bucket. The buckets were kept at room temperature (21–23 °C) in a chamber with artificial light (providing a day-night cycle of 12 h/12 h). After, the dividers were set at the boundary between the test and control substrates and earthworms were counted in both halves. Earthworm avoidance was calculated using Eq. (2).

2 $$A=\frac{C-T}{N}\times 100$$

where *A* is the percentage of avoidance, *C* is number of earthworms in control substrate, *T* is number of earthworms in test substrate, and *N* is the total number of earthworms.

#### Toxicity tests

A phytotoxicity test on white mustard (*Sinapis alba* L.) and an acute toxicity test on aquatic bacteria were performed. In both tests, extracts of degraded 100% C, 10%, 50%, 90%, and 100% gRMW were tested. The extracts were prepared by drying the mixtures of the composted material in an oven at 60 °C for 24 h and finely grinding them. The material was then mixed with distilled water for the phytotoxicity test and with 2% NaCl for the aquatic bacteria test to obtain a concentration of 2 g/L (Hoekstra et al. 2002). The prepared suspensions were shaken on an orbital shaker at 300 rpm for 2 h and then filtered through a GF/C filter (Whatman®, UK). The filtrates were further used in the tests.

The phytotoxicity test was adapted from SIST EN 16086–2:2012. *S. alba* was used due to higher sensitivity to phytotoxic effects. Twenty-five millilitres of filtrate was used per test plate, while the control consisted of 25 mL of distilled water. Ten white *S. alba* seeds were added per plate. The experiment was performed in triplicate. After three days of germination and root growth at room temperature (22 ± 2 °C), the number of germinated seeds was counted and the length of roots was measured. The germination index (GI) was calculated using Eq. (3).

3 $$\mathrm{GI\;}\left(\mathrm{\%}\right)=\frac{{\text{Av}}.\mathrm{\;root\;lenght\;in\;SAMPLE\;}\times {\text{Av}}.\mathrm{\;no}.\mathrm{\;of\;germinated\;seeds\;in\;SAMPLE}}{{\text{Av}}.\mathrm{\;root\;lenght\;in\;CONTROL\;}\times\;{\text{Av}}.\mathrm{\;no}.\mathrm{\;of\;germinated\;seeds\;in\;CONTROL}}\times\;100$$

The acute toxicity test on aquatic luminescent bacteria *Aliivibrio fischeri* was conducted in accordance with ISO 11348–2:2007. Liquid-dried bacteria (LUMIStox LCK 482; Hach Lange GmbH, Germany) were rehydrated and combined with test solutions in a 1:1 ratio. Bioluminescence was measured with the Dr. Lange LUMIStox 300 luminometer (Hach Lange GmbH, Germany) at time 0 and after 30 min. The percentage of bioluminescence inhibition was determined by comparing the response of a negative control solution (2% NaCl) with that of the samples.

The statistical significance of the results of both toxicity tests was analysed by a variance test (ANOVA) with a significance threshold of 0.05, followed by a *t*-test in Excel (Microsoft, USA).

## Results

### Degradation test

**Fig. 1:**
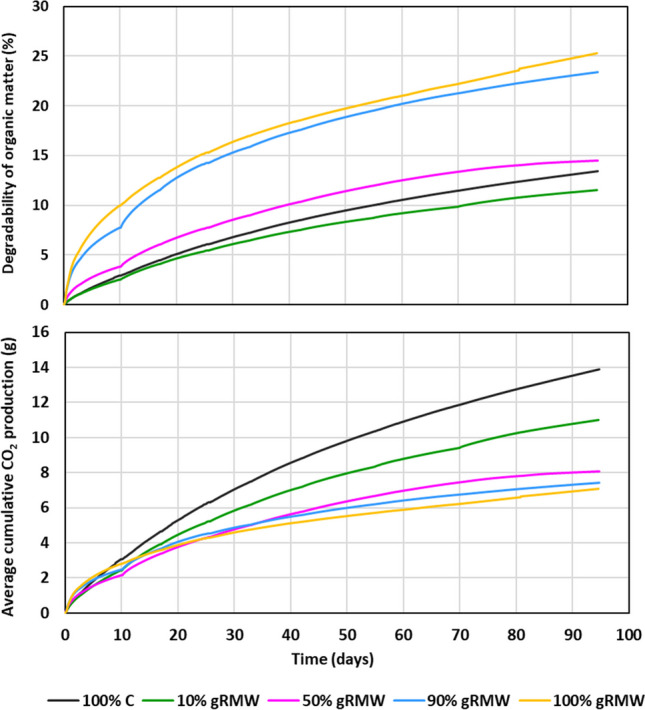
Average cumulative CO_2_ production and organic matter degradability of different mixtures of gRMW and compost during the respirometer degradation test

Degradation test in the respirometer showed that the CO_2_ production was highest in the sample with 100% C and decreased with increasing gRMW content (Fig. 1), which means that more degradable organic matter is presented in compost. However, the degradability of organic matter was inverse of CO_2_ production: the rate of degradability was the highest in the case of 100% gRMW and gradually decreased with increasing compost content, which means that the organic matter in the gRMW was more biodegradable than the organic matter in the compost. Sanitation of degraded material was achieved; results can be found in the Supplementary Information.

### Physical and chemical characterisation

#### Content of volatile matter/ash

The ash content of the samples before and after the decomposition test showed that the relative content of volatile matter increased as the content of gRMW decreased, which was expected. gRWM contains ~ 14% volatile matter (ignition at 550 °C presents raw organic matter), of which ~ 4–5% comes from the original structure of RMW (organic adhesives), while ~ 10% comes from the roots of plants growing in greenhouses (Šubic et al. 2021). In the decomposition test, ~ 1–3% of the total gRMW was degraded, corresponding to 25% of the organic matter (Fig. 1).

#### Water holding capacity

**Fig. 2:**
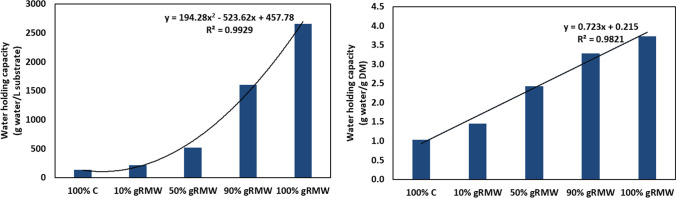
Water holding capacity of the different mixtures of gRMW and compost in grams of water per litre of substrate (left) and grams of water per gram of dry substrate (right)

Water holding capacity increased with increasing content of gRMW in the sample (Fig. 2). At 100% gRMW, 1 g dry weight of the sample retained just over 3.5 g of water.

#### pH, electrical conductivity, carbon, and nutrients

Chemical analyses of gRMW and compost mixtures showed that gRMW was slightly acidic (pH = 6.4) and that the addition of slightly alkaline compost neutralised the mixtures (Table 1). Interestingly, EC was the highest in 90% gRMW, although both ‘pure’ substrates, 100% C and 100% gRMW, had similar and relatively low EC of 1.4–1.5 mS/cm. Mineral N fractions (N_min_; NH_4_-N and NO_3_-N) were also the highest in 90% gRMW, while 100% C had low N_min_ concentrations despite the highest N-total content. Degradation reduced N_min_ concentrations by 70% (90% gRMW) to 90% (10% gRMW) for both NH_4_-N and NO_3_-N; however, the percent N-total to dry weight remained about the same before and after degradation. The reduction of NH_4_-N was probably due to nitrification, while the reduction of NO_3_-N under the aerobic conditions was most likely due to leaching rather than denitrification. In general, N_min_ content in gRMW with or without compost addition was 20–30 times higher compared to arable soils. In detail, explanation of N_min_ results is provided in Supplementary Information.

**Table 1:** pH, electrical conductivity (EC), carbon and nutrients (measurements in extracts of solid substrates), and metallic elements (total element content in mg/kg dry weight) in samples before and after the aerobic degradation test (*NH*_*4*_*-N* ammonia nitrogen, *NO*_*3*_*-N* nitrate nitrogen, *N*_*min*_ total mineral nitrogen, *AL-P*_*2*_*O*_*5*_ plant-available phosphorus, *AL-K*_*2*_*O* plant-available potassium, *C*_*org*_ organic carbon, *C*_*org*_*/N* ratio between organic C and total N). Values before degradation test are results of the analyses of composite samples of different compost and gRMW mixtures, while values after degradation test are average and standard deviation of two or three reactor vessels in the respirometer

		Before degradation test					After degradation test					Limit. val.*
		100% C	10% gRMW	50% gRMW	90% gRMW	100% gRMW	100% C	10% gRMW	50% gRMW	90% gRMW	100% gRMW	
pH		7.0	7.1	6.9	6.6	6.4	7.4 ± 0.1	7.4 ± 0.1	7.1 ± 0.1	6.5 ± 0.1	6.4 ± 0.2	/
EC	mS/cm	1.22	1.48	3.08	2.66	3.47	1.9 ± 0.8	2.0 ± 0.03	2.4 ± 0.3	2.8 ± 0.2	1.4 ± 1.1	/
NH_4−_N	mg/100 g	0.8	1.3	13	18.5	24.4	1.1 ± 0.2	0.6 ± 0.1	2.4 ± 0.3	11.1 ± 0.9	8.4 ± 5.7	/
NO_3−_N	mg/100 g	60	251	251	251	252	9.3 ± 8.8	28 ± 2.0	56 ± 1.5	75 ± 2.5	36 ± 32	/
N_min_	mg/100 g	61	253	264	270	276	10 ± 8.8	29 ± 1.9	58 ± 1.8	86 ± 1.6	44 ± 38	/
AL-P_2_O_5_	mg/100 g	360	361	356	375	369	314 ± 2	307 ± 0.4	291 ± 11	307 ± 5	313 ± 2	/
AL-K_2_O	mg/100 g	617	615	415	253	219	494 ± 246	377 ± 518	410 ± 41	285 ± 52	156 ± 53	/
C-total	[%]	18.3	16.6	11.1	7.1	6.6	16.6 ± 0.7	15.2 ± 0.04	10.6 ± 0.01	6.9 ± 1.2	6.3 ± 0.2	/
N-total	[%]	1.77	1.64	1.21	0.97	0.92	1.60 ± 0.01	1.57 ± 0.01	1.20 ± 0.00	0.98 ± 0.11	0.71 ± 0.19	/
Carbonate-C	[%]	2.09	1.74	1.19	0.35	0.30	2.09 ± 0.11	1.74 ± 0.09	1.19 ± 0.06	0.35 ± 0.02	0.3 ± 0.02	/
C_org_	[%]	16.2	14.9	9.9	6.7	6.3	14.5 ± 0.8	13.5 ± 0.7	9.4 ± 0.5	6.5 ± 0.3	6.0 ± 0.3	/
C_org_/N		9.1	9.1	8.2	6.9	6.8	9.1 ± 0.8	8.6 ± 0.7	7.8 ± 0.5	6.7 ± 0.5	8.5 ± 0.5	/
Ca	[%]	6.6	7.9	9.2	11.2	12.8	6.1 ± 0.4	7.5 ± 0.2	10.5 ± 0.4	13.5 ± 0.3	13.7 ± 1.3	/
Mg	[%]	1.32	1.82	2.86	3.78	4.45	1.34 ± 0.03	2.01 ± 0.05	3.21 ± 0.08	4.31 ± 0.02	4.52 ± 0.40	/
Na	[%]	0.12	0.36	0.91	1.42	1.59	0.10 ± 0.02	0.47 ± 0.02	1.00 ± 0.03	1.46 ± 0.01	1.48 ± 0.14	/
Fe	[%]	1.63	1.94	2.51	3.22	3.85	1.72 ± 0.14	2.20 ± 0.06	3.00 ± 0.14	3.87 ± 0.23	4.10 ± 0.15	/
Al	[%]	1.03	1.83	3.63	5.52	6.90	0.99 ± 0.02	2.34 ± 0.01	4.54 ± 0.23	6.86 ± 0.13	7.40 ± 0.58	/
K	[%]	0.77	0.72	0.51	0.42	0.42	0.70 ± 0.12	0.80 ± 0.01	0.60 ± 0.04	0.45 ± 0.01	0.38 ± 0.05	/
P	[%]	0.71	0.72	0.61	0.61	0.75	0.63 ± 0.02	0.69 ± 0.02	0.75 ± 0.01	0.75 ± 0.09	0.81 ± 0.01	/
S	[%]	0.29	0.23	0.20	0.18	0.17	0.23 ± 0.05	0.24 ± 0.05	0.14 ± 0.00	0.09 ± 0.04	0.06 ± 0.01	/
Mo	mg/kg	2.5	10.2	18.7	31.2	32	2.1 ± 0.6	5.9 ± 0.1	12.7 ± 0.6	20.4 ± 1.3	15.1 ± 5.5	/
Cu	mg/kg	288	89.0	58.6	45.9	46.7	97.2 ± 4.4	87.6 ± 2.3	59.5 ± 1.3	46.2 ± 1.3	44.0 ± 0.3	300
Pb	mg/kg	55.3	37.9	20.3	3.9	2.8	50.1 ± 5.2	38.8 ± 0.4	17.8 ± 0.2	4.5 ± 0.6	2.8 ± 0.2	120
Zn	mg/kg	335	260	159	103	92	321 ± 16	256 ± 0.0	165 ± 1	110 ± 11	113 ± 2	800
Ni	mg/kg	23.4	87.9	97.9	144.7	116.7	23.0 ± 0.4	21.6 ± 0.6	18.2 ± 0.1	19.0 ± 1.5	18.0 ± 0.7	100
Mn	mg/kg	831	1409	2734	4191	4744	799 ± 42	1725 ± 50	3294 ± 158	4829 ± 236	5171 ± 258	/
Cd	mg/kg	0.7	0.8	0.5	0.2	0.1	0.9 ± 0.1	0.7 ± 0.1	0.4 ± 0.1	0.2 ± 0.0	0.2 ± 0.1	1.5
Cr	mg/kg	33	296	799	1259	1470	33 ± 2.1	326 ± 8.5	830 ± 43	1282 ± 30	1404 ± 25	/
Hg	mg/kg	0.21	0.12	0.08	0.02	0.03	0.22 ± 0.07	0.17 ± 0.05	0.07 ± 0.00	0.01 ± 0.00	< 0.01	1.00
Se	mg/kg	< 0.5	< 0.5	< 0.5	1.1	< 0.5	0.67 ± 0.21	0.75 ± 0.35	0.65 ± 0.07	0.60 ± 0.14	< 0.5	/
As	mg/kg	5.8	4.4	3.0	1.6	1.9	5.7 ± 0.5	4.9 ± 0.0	3.1 ± 0.1	1.8 ± 0.2	1.6 ± 0.2	40

*Limiting values according to EU Regulation 2019/1009

Similarly, plant-available AL-P_2_O_5_ was 20 times higher than in fertile arable soils. Its content decreased slightly during degradation test and did not differ significantly in any of the treatments. AL-K_2_O was three times higher in 100% C than in 100% gRMW, but the latter was still five to ten times richer than fertile arable soil. During the degradation test, AL-K_2_O decreased by 20 to 30% in compost and 100% gRMW, while it increased by 13% in 90% gRMW.

One hundred percent gRMW contained 2.5% carbonates (or 0.3% carbonate-C), which could be accumulated in the RMW during the tomato growing season. Mixing with more carbonate-rich compost proportionally increased the carbonate-C content in the samples.

The compost we used was already quite mineralized, containing just 16.2% and 14.5% organic C (C_org_) before and after the degradation test, respectively. Compared to the compost, the 100% gRMW was poor in C_org_, at only 6.3 and 6.0% before and after the degradation test, respectively. This was expected and was due to organic adhesives and plant root residuals. However, 6% C_org_ is the concentration normally found in organic-rich mineral soils (de Brogniez et al. 2015).

Secondary macronutrients Ca, Mg, and Na were high in 100% gRMW. Degradation did not result in significant changes in these concentrations—in the case of 100% C, there was a slight loss of Ca and Na, but in the case of 90% and 50% gRMW, the proportions increased after degradation. Because of the high concentration of these elements, which are considered alkaline cations, one would expect the gRMW to be pH neutral or even slightly basic, but it was slightly acidic, probably due to acidifying oxidation of ammonia to nitrate.

The percent K and S were low in gRMW, while the P content was similar in both compost and gRMW, suggesting that gRMW may be a good P source. In the case of 100% gRMW, all three elements decreased during the degradation test, while K and P increased in 90% and 50% gRMW, respectively.

#### Micronutrients and other elements

Concentrations of micronutrients and other non-nutritional elements in compost and in 100% gRMW were mostly in the same range. The exception was Cr, present in 40 times higher concentration in 100% gRMW as compared to 100% C (Table 1). Mo was high in gRMW (30 mg/kg), but about 50% was lost during degradation. gRMW had low Cu and Pb content, while Ni content was just above the limit before degradation; however, after degradation, most of Ni was lost most likely due to leaching. One hundred percent gRMW had lower Zn content than compost, both, before and after degradation. According to EU Regulation 2019/1009 for EU fertilising products, the pollutant limits (Cd, Cr(VI), Hg, Ni, Pb, inorganic As, Cu, and Zn) were not exceeded in all of the degraded samples.

#### Cr(VI)

Concentrations of total Cr(VI) in undegraded 100% gRWM and in degraded 90%, 50%, and 100% gRMW samples determined by the HPLC-ICP-MS and spectrophotometry are presented in Table 2.

**Table 2:** Concentrations of total Cr(VI) (dry weight) in 100% gRWM sample before the degradation and in degraded gRMW samples determined by HPLC-ICP-MS and spectrophotometry. Measurement uncertainty for HPLC-ICP-MS was lower than ± 3%, while for spectrophotometry lower than ± 5%

	Total Cr(VI) (mg/kg)	
	HPLC-ICP-MS	Spectrophotometry
100% gRMW before degradation	0.13 ± 0.02	< 1
100% gRMW after degradation	< 0.060	2.76
90% gRMW after degradation	0.12 ± 0.02	4.35
50% gRMW after degradation	< 0.060	5.47
Limiting value*	2	2

*EU Regulation 2019/1009

Data from spectrophotometric determination indicate that the technique overestimates Cr(VI) concentration due to its liability to interferences caused by coloured substances of sample matrix (compost and gRMW), which absorb at the wavelength for quantification of the formed Cr magenta complex (540 nm). These interferences may lead to a significant overestimation of Cr(VI) concentrations in measured samples (Milačič and Ščančar 2020). Limit of detection (LOD) for this method was 1 mg/kg Cr(VI) and limit of quantification (LOQ), 3.33 mg/kg, which was above the permissible limit specified in the legislation (2 mg/kg Cr(VI)).

The results obtained by the HPLC-ICP-MS indicate that Cr(VI) concentrations in gRMW samples were very low. In 100% gRMW before degradation, Cr(VI) concentration was 0.13 mg/kg, while after degradation, it was reduced to below LOD (< 0.060 mg/kg). Interestingly, in 90% gRMW after degradation, Cr(VI) was only slightly reduced, while in 50% gRMW sample, Cr(VI) concentration was again below LOD. The accuracy of the method was confirmed by spike recovery tests. To check for potential species interconversion during the analytical procedure, 100% gRMW sample was doubly spiked with ^50^Cr(VI) and ^53^Cr(III). More information on LOD and LOQ determination of both methods, typical chromatograms of Cr(IV) obtained with HPLC-ICP-MS method, spike recovery test, and species interconversion can be found in Supplementary Information.

The Cr(VI) concentrations in gRMW samples determined by the HPLC-ICP-MS were far below the legislative regulations 2 mg/kg (EU Regulation 2019/1009), while the spectrophotometric method gave false overestimated values. Based on the results from the accuracy check and species interconversion test, considering also low LOD and LOQ values, we can conclude that the HPLC-ICP-MS is highly sensitive method, providing accurate and reliable results for the determination of Cr(VI) in complex samples, and can be recommended as a method of choice for the determination of Cr(VI) in gRMW samples.

#### Legislative aspects

EU Regulation 2019/1009 lays down the rules on the making available on the market of EU fertilising products, which, amongst others, specifies limiting values of specific contaminants in inorganic and organic soil amendments. Concentrations of Cd, Hg, Ni, Pb, inorganic As, Cu, and Zn were not exceeded in degraded samples (Table 1). Despite high total Cr content (Table 1), the content of Cr(VI) (Table 2) was far below the limiting value of 2 mg/kg all samples. Therefore, elements regulated by the EU legislation as well as Cr(VI) do not represent an environmental hazard.

### Bioassays to evaluate the ecological hazard

#### Avoidance test with earthworms

**Fig. 3:**
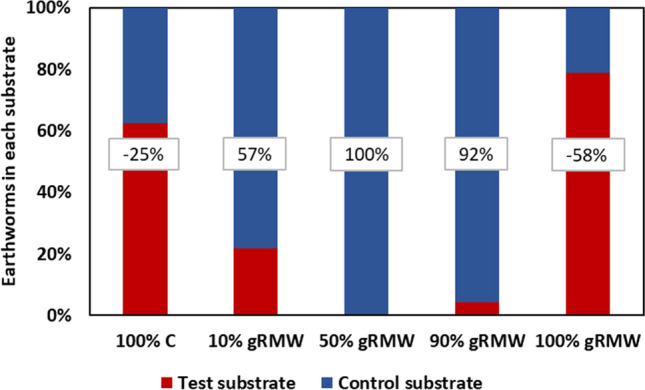
Percentage of earthworms in the test and control substrates and percentage of avoidance (in grey boxes; positive numbers indicate avoidance and negative numbers preference) at the end of the avoidance test (48 h)

The avoidance test is a method by which we can represent the habitat function based on the avoidance of organisms of test media. Positive values indicate that earthworms avoid the test substrate, while negative values indicate a preference for that substrate. Substrates with less than 20% of earthworms are considered to have limited habitat function (ISO 17512–1:2008). The results show that earthworms avoided mixed substrates (e.g., 10%, 50%, and 90% gRMW) (Fig. 3). Avoidance was high or complete for the 50% and 90% gRMW and lower for 10% gRMW. No avoidance was observed in 100% C treatment, which was expected for the control treatment. No avoidance was observed also in 100% gRMW (− 58%). This was not expected since the avoidance of 50% gRMW and 90% gRMW was high. Therefore, the experiment was repeated with this treatment with another three replicates, but the results showed a similar trend (− 55% and − 58% in the first and second experiments, respectively).

#### Toxicity tests

**Fig. 4:**
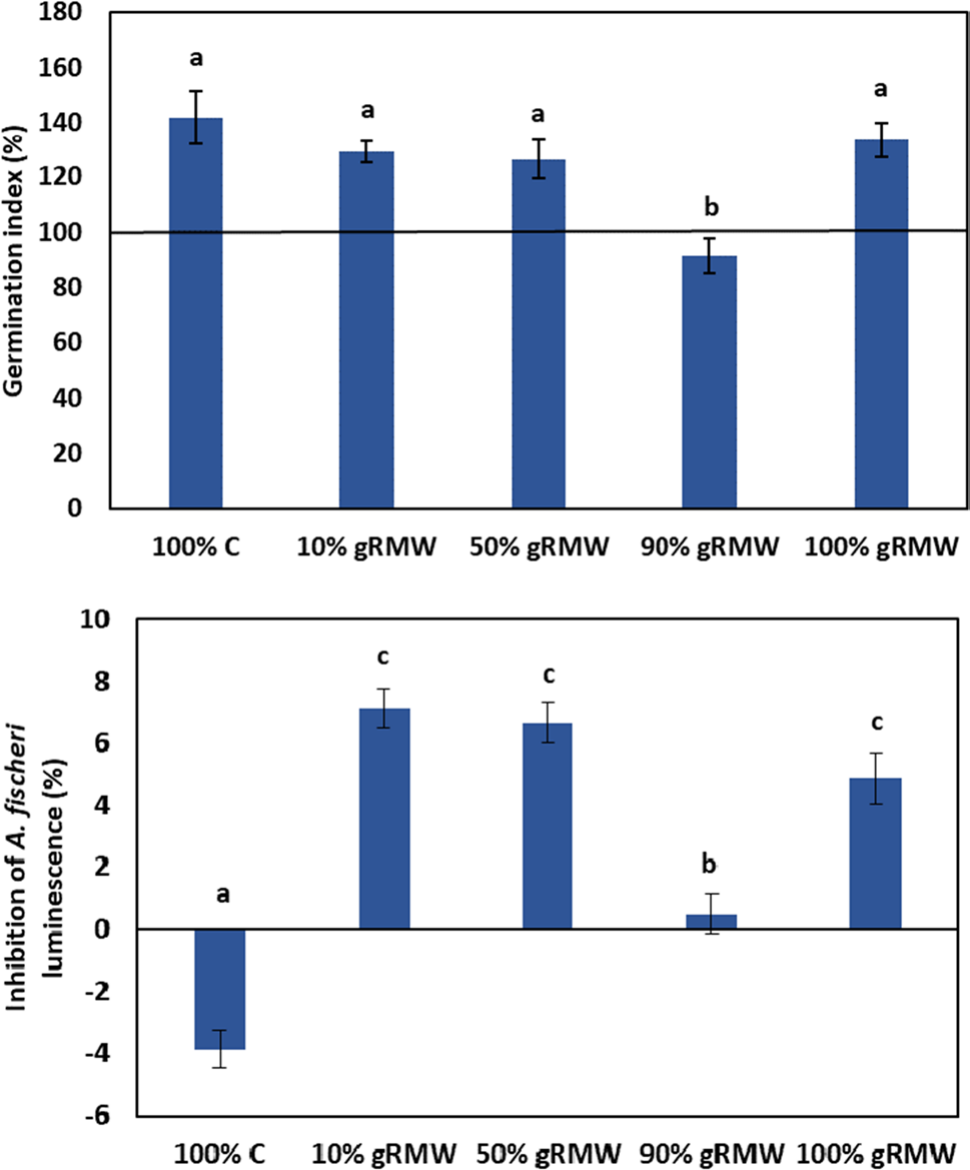
Phytotoxicity test on *S. alba* L. (*n* = 3 ± SD; germination index of distilled water is 100%) (top) and inhibition of bioluminescence (%) of *A. fischeri* (*n* = 6 ± SD) (bottom) in different extracts from composted compost and mineral wool mixtures. Different letters (a, b, c) represent statistically different values, while equal letters mean that there was no statistically significant difference between the values

Phytotoxicity is defined as a delay in seed germination, an inhibition of plant growth, or an adverse effect on plants caused by substances or growing conditions. A germination index (GI) below 80% indicates phytotoxicity, 80 to 120% indicates no phytotoxicity, while GI above 120% indicates a stimulatory effect on plants. The extracts of 100% C, 100% gRMW, 50% gRMW, and 10% gRMW showed stimulatory effect on germination and primordial growth of roots of white mustard (GI > 120%). The extract of 90% gRMW had a slightly lower GI than the control, but above 80% (GI = 92%), indicating that there was no phytotoxic effect (Fig. 4).

The acute toxicity test on *A. fischeri* showed that there was no statistically significant difference between the inhibitions of luminescence in the extracts from 100%, 50%, and 10% gRMW (P > 0.05), after 30 min, and that the inhibition was low (4.9, 6.7, and 7.1%, respectively). Ninety percent gRMW showed no toxicity to *A. fischeri* (inhibition at 0.5 ± 0.6%), while 100% C promoted *A. fischeri* bioluminescence compared to the negative control (2% NaCl). Luminescence in this sample increased by 3.9 ± 0.6%. So, all the suspensions prepared showed low toxicity (< 8%) indicating that gRMW therefore can be applied to soil without foreseeable environmental concerns for aquatic organisms; however further, toxicity tests should be conducted to confirm this.

## Discussion

### Degradation test

Numerous studies have tested various waste materials as additives or bulking agents to improve the degradation of organic matter in the composting process (Barthod et al. 2018; Li et al. 2021; Chen et al. 2023). In fact, the use of gRMW as an additive for composting is already practiced in some countries. Grodan, one of the largest producers of RMW for hydroponics, supports farmers with information on composting options (Grodan 2022); however, no scientific literature has been published on this topic. The results of this study indicate that gRMW has the potential to be composted without being mixed with organic waste or inoculated with compost. Although gRMW has a low organic matter content compared to compost, the organic matter is easily biodegradable.

### Physical and chemical characterisation

gRMW mixtures exhibited exceptional water holding capacity, which is a desirable characteristic of soil amendments. Similarly, biochar-based soil amendments have been reported to increase soil water retention (Günal et al. 2018; Śpitalniak et al. 2021; Kang et al. 2022). The composition of reclaimed RMW largely depends on the composition of the incoming material; however, all manufactured RMW usually comply with Note Q of Regulation EC 1272/2008. Yliniemi et al. (2021), who presented results from more than 70 samples of glass and rock mineral wool used in construction, found that Cr, Ni, Cu, Zn, and Pb are present in both types at low concentrations.

The C_org_/N ratio is an indicator of the availability of substrates and energy (C_org_) versus the availability of N which is a biogenic nutrient. The ratio is in equilibrium for nutrition of soil microorganisms at about 20. At a higher ratio, more C_org_ is available, which results in microbial N immobilization, whereas at ratios below 20, N can accumulate in soil as a physiological microbial surplus and is available for plants. In practice, organic fertilisers are effective in promoting plant growth when the C_org_/N ratio is between 1 and 15 (Brust 2019). The substrates of gRMW and compost had C_org_/N ratio between 6.7 and 9.1, which means they are suitable for rapid release of N for plant uptake. Additionally, plant-available P and K levels were 20 and 5–10 times higher, respectively, than in fertile arable soils, making gRMW a nutrient-rich soil amendment. Furthermore, gRMW is rich in Na, Ca, and Mg. These elements are important for soil quality, with Ca being the main binder for stability of soil structure, as it binds clay and humus colloids through cation bridges. Heavy clay soils have the highest Ca requirement, where 80% of all cation exchange sites should be occupied by Ca^2+^. gRMW as a soil amendment can, therefore, provide Ca in clay soils.

No legislative limits for elements were exceeded in gRMW mixtures, with Mo and Zn present in favourable concentrations. Mo is important for the conversion of nitrates to nitrites and contributes to the synthesis of essential amino acids in plants. In legumes, the relationship between Mo and N contributes to the fixation of atmospheric N in the roots of nodulated rhizobia. Zn is used in the formation of chlorophyll, some carbohydrates, and auxins that contribute to growth regulation and stem elongation, as well as in the conversion of starch to sugars, and its presence in plant tissue helps the plant withstand cold temperatures. The normal range for Zn in plant tissue is 15–60 mg/kg (Mengel and Kirkby 2001).

In the environment, Cr is present mostly in its trivalent, Cr(III), and hexavalent, Cr(VI), forms. Cr(III) originates mainly from geogenic sources, is the most abundant, and has a relatively low toxicity. Cr(VI) is present mainly as an industrial pollutant and is highly toxic. Cr(VI) compounds are carcinogenic, mutagenic, and genotoxic, so their concentrations in water and soil are under environmental scrutiny (Pezennec 2007; Milačič and Ščančar 2020). As demonstrated by our results obtained from the HLPC-ICP-MS analysis, all gRMW mixtures contained Cr(VI) in concentrations far below the legislative limit. Our results also demonstrated the importance of using the reliable analytical methodology for the determination of Cr(VI) concentrations in samples with complex matrices. The spectrophotometric method, specified in ISO 15192:2010, was proven to be unsuitable for coloured samples. Only by the use of reliable analytical methodologies, adequate conclusions on the environmental impacts of the recycled waste materials can be made (Milačič and Ščančar 2020; Ščančar and Milačič, 2014). Marković et al. (2022), demonstrated that the uptake of Cr(III) species in plants is significantly lower than that of Cr(VI) and that the uptaken Cr(VI) was effectively reduced to Cr(III) by polyphenolic and other reducing substances in plants. Based on this knowledge and the results of present research, we conclude that there are no environmental or dietary concerns about amending soil with gRMW.

### Bioassays to evaluate the ecological hazard

Most research on soil amendments includes phytotoxicity studies with various biochars, composts, biosolids, and bioenergy byproducts. The reuse of reclaimed hydroponic growing media, such as gRMW, as a soil amendment is a new concept, so to our knowledge, there are no data on gRMW (phyto)toxicity in the literature. In general, gRMW had no phytotoxic or phytostimulatory effect on germination and early root development in our trials with *S. alba*. Many potential soil amendments have positive to negative effects on germination. For example, Kalderis et al. (2019) obtained strong phytostimulatory effects of biochar from a 50:50 mixture of paper sludge and wheat husks in rocket (*Eruca sativa*) and lettuce (*Lactuca sativa*) (332 and 169%, respectively); however, hydrochar from orange peels exhibited strong phytotoxicity on both plants (GI 0 and 2%, respectively). Visioli et al. (2016) reported biochar from grape pomace and wheat straw exhibited strong phytotoxicity (GI < 60%) to cucumber (*Cucumis sativus* L.), garden cress (*Lepidium sativum* L.), and sorghum (*Sorghum saccharatum*). Phytotoxicity was generally attributed to high EC of soil amendments, presence of phytotoxins (phenols, chemically active functional groups that increase cation exchange capacity), presence of certain ecotoxic metal compounds (Cu and Zn), degree of stability of organic matter, or induced changes in soil pH (Zubillaga and Lavado 2006; Gell et al. 2011; Visioli et al. 2016; Venegas et al. 2018). Although high EC is usually related to negative effect (e.g., lower yields and lower plant biomass), it is worth noting that in some cases, it can exhibit positive eustress on plants. High EC nutrient solution (7 dS·m^−1^) increased dry matter and bioactive compounds (e.g., β-carotene, lutein, and chlorophyll a and b) in hydroponically grown cucumbers (Łaźny et al. 2022). In our case, the gRMW mixtures did not have elevated levels of metallic compounds. The pH and EC of gRMW mixtures were in the ranges of 6.4–7.4 and 1.4–2.8 mS/cm, respectively, which are values that would not likely cause serious changes in these two parameters in soil.

In addition to the phytotoxicity test, we performed a toxicity test based on inhibition of bioluminescence of the bacterium *A. fischeri*. Although this test is not directly relevant to soil application, it served as an additional indicator for evaluating the ecotoxicity of eluates from gRMW. In addition, the toxicity of aqueous extracts represents the potential toxicity that occurs in the environment when the material is soaked during rainfall or irrigation. In this case, leachates could enter water bodies and/or groundwater. The toxicity of aqueous extracts may originate from binders and dissolved byproducts of gRMW that may originate from RMW material or from hydroponic nutrient solution and roots remains. In contrast to RMW used in construction where binders like phenolic resin, polyesters, melamine-urea–formaldehyde, polyamides, and furan-based resin are used (Yliniemi et al. 2021), the RMW horticultural boards are produced with a biobased organic binder. gRMW mixtures showed low toxicity (< 8% inhibition) to *A. fischeri*, which is lower or comparable to values reported in the literature for biochar. Oleszczuk et al. (2013) tested the toxicity of various biochars, from silver grass (*Miscanthus* sp.), coconut shells, willow shoots (*Salix viminalis*) to wheat straw (*Triticum* L.). Most of them were highly toxic to *A. fischeri*, with inhibition values ranging from 40 to 99%. Only biochar derived from coconut shells showed low toxicity of 12% inhibition of luminescence. On the other hand, biochar from mixtures of sewage sludge and willow produced at 500 and 600 °C had no toxicity to *A. fischeri* (Kończak et al. 2020). This demonstrates that the toxicity of biochar varies greatly with the feedstock and the temperature used during pyrolysis. This effect has often been attributed to the presence of pollutants in biochar, such as polycyclic aromatic hydrocarbons and heavy metals; high pH and/or salinity; or a strong ability to adsorb nutrients and thus reduce their availability (Godlewska et al. 2021). Very little to no toxic effect on *A. fischeri* was found for various mixtures of gRMW and compost, so we can rule out the presence of toxic substances at levels likely to cause harm. In addition to the release of toxic substances, the potential risk to freshwater and groundwater from the fine particles (fibres) of RMW can be assessed. As mentioned above, the RMW used in this study complies with Note Q of EC Regulation 1272/2008, which states that it is not carcinogenic to humans and has a European Certification Board Certificate issued by the Belgian Construction Certification Association npo 2023; however, further studies are required to assess the risks to aquatic and terrestrial biota.

In addition to two toxicity tests, we also evaluated the habitat function of gRMW mixtures using an earthworm avoidance test. Earthworms are considered suitable bioindicators of soil pollution because they are in constant contact with the soil and feed on soil particles due to their thin cuticle and chemoreceptors with which they receive stimuli and pollutants all over their body (Paoletti 1999; Ebagnerin Tondoh et al. 2006; Piron et al. 2012). They have been used as indicators of soil contamination with heavy metals (Suthar et al. 2008), pesticides (Yao et al. 2020), and persistent organic pollutants (Shi et al. 2019). Interestingly, gRMW mixtures with compost were unfavourable to earthworms and most of them were found in the control substrate. Therefore, these can be classified as substrates with limited or medium habitat function. However, this trend was not observed in 100% gRMW, where 80% of earthworms were found. This may be explained by the ecology of *L. rubellus*. This earthworm is an epigeic earthworm, meaning that it lives at the soil surface in leaf litter and feeds on it. Thus, *L. rubellus* could find a similar biotope in the mineral wool, which, similar to leaf litter, has a high insulating capacity and pore content.

All in all, the bioassays showed that gRMW mixtures with compost did not exhibit toxicity to plants or aquatic bacteria, but they could have intermediate or limited habitat function for soil-dwelling animals. The opposite was true for gRMW that was not mixed with compost. According to the results of bioassays presented herein, we recommend that further research on the use of gRMW focuses on composting and reusing 100% gRMW, not in combination with compost. To assess the environmental risks, the application of gRMW as a soil amendment should be tested in a controlled environment in lysimeters to account for leaching of potentially toxic substances and RMW fines.

The recommendations for composting of gRMW and its further use can be found in the Supplementary Information.

## Conclusions

This work has shown that recovered RMW from hydroponics has the potential to be used as a soil amendment and thus can be converted from waste to a value-added product. The organic matter in gRMW exhibited a higher rate of decomposition and lower cumulative CO_2_ production compared to compost. Therefore, composting of sole gRMW without mixing with inoculant compost is recommended, which simplifies the production because growers can produce it themselves and avoids the potential risk of secondary pollution from municipal compost. Degraded gRMW exhibited parameters of a safe soil amendment according to EU regulation and showed no adverse effects on the organisms tested. Composting process and end products could vary when gRMW is mixed with compost (i.e., mixing gRMW with compost triggers different processes during composting than composting pure mineral wool). Due to its low organic matter content, gRMW could also be used in non-composted form as a soil amendment; however, in this case, sterilisation/pasteurisation should be carried out prior to application. Future research on gRMW should focus on field testing, upscaling, long-term exposure, and resulting potential chronic toxicity and environmental effects.

### Supplementary Information

Below is the link to the electronic supplementary material.

Supplementary file1 (DOCX 168 KB)

## Data Availability

Data will be made available on request.
