# Supplementary material for: Composting of recovered rock wool from hydroponics for the production of soil amendment
Source: Environ Sci Pollut Res Int. 2024 Apr 4;31(20):29280–93. doi: 10.1007/s11356-024-33041-2 (PMC11058614; doi:10.1007/s11356-024-33041-2)
Supplement: Supplementary file 1 — Supplementary file1 (DOCX 168 KB) [file 11356_2024_33041_MOESM1_ESM.docx]

*Environmental Science and Pollution Research*

**Composting of recovered rock wool from hydroponics for the production of soil amendment**

Darja Istenič^a,b*^, Franja Prosenc^a,c^, Neva Zupanc^d^, Matejka Turel^e^, Andrej Holobar^e^, Radmila Milačič^f^, Stefan Marković^f^, Rok Mihelič^g^

^a^Faculty of Health Sciences, University of Ljubljana, Zdravstvena pot 5, Ljubljana, Slovenia; [darja.istenic@zf.uni-lj.si](mailto:darja.istenic@zf.uni-lj.si)

^b^Faculty of Civil and Geodetic Engineering, University of Ljubljana, Jamova cesta 2, Ljubljana, Slovenia;

^c^BioResource Systems Research Group, School of Civil Engineering, University of Leeds, Woodhouse Lane, Leeds LS2 9JT, UK; [f.prosenc@leeds.ac.uk](mailto:f.prosenc@leeds.ac.uk)

^d^Knauf Insulation d.o.o., Trata 32, Škofja Loka, Slovenia; [neva.zupanc@knaufinsulation.com](mailto:neva.zupanc@knaufinsulation.com)

^e^ECHO Instruments d.o.o*.,* Zeče 25, Slovenske Konjice, Slovenia; [matejka@echo.si](mailto:matejka@echo.si), andrej@echo.si

^f^Department of Environmental Sciences, Jožef Stefan Institute, Jamova cesta 39, Ljubljana, Slovenia; [radmila.milacic@ijs.si](mailto:radmila.milacic@ijs.si), [stefan.markovic@ijs.si](mailto:stefan.markovic@ijs.si)

^g^Biotechnical Faculty, University of Ljubljana, Jamnikarjeva 101, Ljubljana, Slovenia, [rok.mihelic@bf.uni-lj.si](mailto:rok.mihelic@bf.uni-lj.si)

*corresponding author

***SUPPLEMENTARY INFORMATION***

# SUPPLEMENTARY MATERIALS AND METHODS

## Starting material

Horticultural rock mineral wool (RMW) is made of various mineral mixtures, is light, dimensionally stable, and ensure high water retention and air ratio. It is used in professional horticulture, especially for hydroponic vegetable growing. The RMW horticultural boards investigated in this study were manufactured by Knauf Insulation d.o.o. according to a standard process that complies with Note Q of European Regulation No. 1272/2008, which means that they are not classified as carcinogenic. The non-carcinogenicity of RMW has also been confirmed by other authors (Barly et al., 2019; Okhrimenko et al., 2022).

Different preparation processes of samples of RMW from greenhouses (gRMW) were used in different bioassays throughout the study. The avoidance test with earthworms (*Lumbricidae*) was performed on the degraded gRMW after the degradation test. The acute toxicity test with *Aliivibrio fischeri* and the phytotoxicity test with white mustard (*Sinapis alba* L.) were performed on the extracts prepared from the degraded gRMW.

## Degradation test

Degradation test was performed in respirometer ER12 produced by ECHO Instruments Ltd. More details can be found here: https://echoinstruments.eu/respirometers/respirometer/.

The temperature in the respirometer was set to 58 °C in order to achieve sanitation of the samples. This was tested by determining the presence of *Salmonella* spp. And number of *E. coli* before and after the degradation tests according to the standards ISO 6887-1:2017, ISO 16649-2:2001 and ISO 6579-1:2017. The initial air flow through the reactors was set to 0.5 L/min. After a few days of measurements, the flow was reduced to 0.05 L/min in all reactors, except in the case of 100% gRMW, where it was set to 0.1 L/min due to significant moisture in the gRMW.

## Physical and chemical characterisation

The pH measurements were performed according to ISO 10390:2005. Measurements were performed electrometrically using a pH meter in a sample suspension. 10 g of the sample was weighed and 100 mL of 0.01 M CaCl_2_ solution was added. The sample was mixed well and allowed to stand for 2 h, after the pH was measured in the supernatant.

Electrical conductivity (EC) was measured according to ISO 11265:1996. The EC was measured in the extraction solution using a conductometer MA 5966 (Iskra, Slovenia). 10 g of the sample was weighed and distilled water was added at a ratio of 1:10. The sample was then mixed well to soak the entire content. The resulting suspension was immediately filtered through filter paper and EC measured in the extraction solution.

The content of total carbon (C-total) and total nitrogen (N-total) was determined according to ISO 10694:1996 using the organic elemental analyser Vario MAX (Elementar UK Ltd., UK), which operates on the principle of dry incineration. 1 g of sample was weighed, and after incineration at 900 °C, the contents of C and N were determined. The amounts of the elements were expressed in weight percent; the organic C was calculated by subtracting the carbonate C from the total C. Carbonate C was determined by the volumetric method as per ISO 10693:1995.

For the analysis of iron (Fe), aluminium (Al), molybdenum (Mo), copper (Cu), lead (Pb), zinc (Zn), nickel (Ni), manganese (Mn), arsenic (As), cadmium (Cd), calcium (Ca), phosphorous (P), chromium (Cr), magnesium (Mg), natrium (Na), potassium (K), mercury (Hg), sulphur (S), selenium (Se), the subsamples of 0.5 g were leached in hot Aqua Regia and elements determined by inductively coupled plasma mass spectrometry (ICP-MS) following the BS EN 13650:2001. The analysis was performed by Bureau Veritas Commodities Canada Ltd (in-house laboratory analysis package, code: AQ200).

Cr(VI) in gRMW samples was determined in sample extracts, which were prepared according to the ISO 15192:2010. 0.5 g of pulverized sample was weighed into a glass beaker and 45 mL of 2% NaOH + 3% Na_2_CO_3_ extraction solution was added. Then, 5 mL of 4 M MgCl_2_ was added in order to prevent oxidation of Cr(III) during the extraction procedure. The sample was transferred to an ultrasonic bath at 90 °C, and the contents were sonicated for 1 h at 700 W. The sample was then cooled to room temperature, filtered through a 0.45 µm membrane filter and diluted 10-times with MilliQ water.

# SUPPLEMENTARY RESULTS

## Degradation test

Sanitation of the degraded material was achieved; *Salmonella* spp. and *E. coli* were present in all samples before the degradation test, while after degradation *Salmonella* spp. was not detected and *E. coli* was detected in less than 10 colony forming units per 10 g sample, which is well below the limit of 1000 colony forming units per 1 g sample set in the EU regulation on fertilizing products (2019/1009).

## Physical and chemical characterisation

### pH, electrical conductivity, carbon and nutrients

It appears that 10% addition of compost to the gRWM stimulated the degradation and mineralization process more than in other treatments, resulting in higher EC. The same observation was confirmed by the concentration of mineral N fractions (N_min_), which were also highest at 90% gRMW. Interestingly, 100% C had low N_min_ concentration, although total N content was highest among treatments. It became clear that the ratio C_org_/N-total is closely correlated to nitrogen mineralization; the lower the ratio, the higher the N_min_ content. The N_min_ concentration in 90% gRMW was quite high compared to rich arable soils, where as little as 2.5 mg NO_3_-N/100 g is considered sufficient for intensive vegetative plant growth (e.g., corn) (Shapiro and Bavougian, 2017); 90% gRMW with a C_org_/N ratio of 6.7 exceeded the recommended agronomic N_min_ content by a factor of 30.

Nitrogen losses are a well-known problem in composting (Hoang et al., 2022). In our case, there was a decrease in mineral N during the degradation test, which was lowest (although still 70%) in 90% gRMW, and the loss of total N was as high as 23% (100% gRMW). gRMW has a high value of mineral nitrogen (mainly in the form of NO_3_-N), so special care should be taken to avoid nitrate losses by leaching.

### Cr(VI)

**Limits of detection and quantification**

Limits of detection (LOD) and quantification (LOQ) for the determination of total Cr(VI) in gRMW samples by spectrometry was calculated based on 3-times or 10-times the standard deviation (*s*) of the signal of blank sample (2% NaOH + 3% Na2CO3 extracting solution), respectively. The LOD of Cr(VI) in 10-times diluted alkaline extract was 1 mg/kg and LOQ, 3.33 mg/kg.

The LOD and LOQ for the determination of total Cr(VI) concentration in gRMW samples by the HPLC-ICP-MS method were calculated as the concentration that provided a signal equal to 3*s* or 10*s* of the blank sample, respectively. The LOD for Cr(VI) in 10-times diluted alkaline extract was 0.060 mg/kg and LOQ 0.200 mg/kg.

**Typical chromatograms for separation of Cr(VI) using HPLC-ICP-MS procedure**

Typical chromatograms of Cr(VI) natural abundance standard solution (10 ng/mL ^nat^Cr(VI)) in alkaline extract (0.2% NaOH + 0.3% Na_2_CO_3_ + 0.04 M MgCl_2_, pH 12) along with a chromatogram of a blank sample (0.2% NaOH + 0.3% Na_2_CO_3_ + 0.04 M MgCl_2_, pH 12) and a chromatogram of 100% gRWM before degradation in 10-times diluted alkaline extract (0.2% NaOH + 0.3% Na_2_CO_3_ + 0.04 M MgCl_2_, pH 12) obtained by the HPLC-ICP-MS procedure, recorded at *m/z* 52 are presented in Fig. S1.

**Fig. S1** Typical chromatograms of ^nat^Cr(VI) standard solution (10 ng/mL Cr) in alkaline extract, a chromatogram of 100% gRWM before degradation in alkaline extract, and a blank alkaline extract obtained by the HPLC-ICP-MS procedure, recorded at m/z 52.

These data indicate that Cr(VI) in standard solution is eluted between 420 and 470 s, with a maximum at 423 s, and that sample 100% gRWM before degradation contains very low concentration of hexavalent Cr, which corresponds to 0.13 ± 0.02 mg/kg Cr(VI).

**Quality assurance**

The accuracy of the HPLC-ICP-MS analytical procedure used for the determination of Cr(VI) was verified by the spike recovery test. In this experiment, hot alkaline extraction, as described in Materials and Methods, was performed on a 100% gRWM sample before degradation to determine the Cr(VI) concentration. To another sample aliquot, 0.1 mL of ^nat^Cr(VI) standard solution with concentration 50 mg/L was added and hot alkaline extraction was performed. The concentration of Cr(VI) in the samples without and with the addition of ^nat^Cr(VI) was then determined in 10-times diluted extracts using the HPLC-ICP-MS procedure. The concentration of ^nat^Cr(VI) added to 100% gRWM sample was 10 mg/kg. The recovery was calculated as the ratio between the determined and expected Cr(VI) concentration. The results are presented in Table S1.

**Table S1:** Spike recovery test. 100% gRWM sample was spiked with 10 mg/kg Cr(VI) and Cr(VI) determined by the HPLC-ICP-MS procedure.

| Sample | Cr(VI) present  (mg/kg) | Cr(VI) added  (mg/kg) | Cr(VI) expected  (mg/kg) | Cr(VI) determined  (mg/kg) | Recovery  (%) |
| --- | --- | --- | --- | --- | --- |
| gRMW 100%  before degradation | 0.13 ± 0.02 | 10.0 ± 0.3 | 10.1 ± 0.3 | 9.7 ± 0.3 | 96 |

Good agreement between the determined and expected concentration was obtained (recovery 96%), confirming the accuracy of the analytical procedure applied.

**Species interconversion test**

To check for possible species interconversion during the extraction procedure, 100% gRWM sample in alkaline extract (2% NaOH + 3% Na_2_CO_3_ + 0.4 M MgCl_2_, pH 12) was doubly spiked with 0.1 mL of ^50^Cr(VI) and 0.1 mL of ^53^Cr(III) enriched isotopic solution, both containing 30 mg/L Cr, and hot alkaline extraction was performed. The speciation analysis was applied in 10-times diluted extracts using the HPLC-ICP-MS procedure, recording the eluted Cr species eluted at *m/z* 50, 52, and 53. The concentration of ^50^Cr(VI) and ^53^Cr(III) added to 100% gRWM, was in 10-times diluted alkaline extract 6 ng/mL Cr. To account for potential Cr(VI) reduction, during the alkaline extraction procedure, ^50^Cr(VI) solution with concentration 6 ng/mL prepared in 0.2% NaOH + 0.3% Na_2_CO_3_ + 0.04 M MgCl_2_ (pH 12) was also injected on the column and the HPLC-ICP-MS procedure applied. The elution profile of ^50^Cr(VI) was recorded at *m/z* 50. The chromatograms of this experiment are presented in Fig. S2.

|  |  |
| --- | --- |

**Fig S2** Chromatograms of Cr species (A) ^50^Cr(VI) spiking solution in 10-times diluted alkaline extract (6 ng/mL Cr), obtained by the HPLC-ICP-MS, recorded at m/z 50, and (B) chromatograms of 100% gRWM sample, doubly spiked with ^50^Cr(VI) and ^53^Cr(III) (6 ng/mL Cr) in 10-times diluted alkaline extract, after the hot alkaline extraction procedure. The chromatograms obtained by the HPLC-ICP-MS were recorded at m/z 50, 52 and 53.

Fig. S2(B) shows that a small peak of Cr(VI) is present in the alkaline extract of the 100% gRWM sample recorded at *m/z* 52 (note that the peak recorded for Cr(VI) at *m/z* 52 is related to the concentration of Cr(VI) in the 100% gRWM sample and the impurity contribution (3.40%) of ^52^Cr(VI) in the ^50^Cr(VI) isotopically spiked solution with a concentration of 10 ng/mL). Furthermore, it can be seen that the ^53^Cr(VI) peak was not detected. This means that the added ^53^Cr(III) was not oxidized during the alkaline extraction procedure. It was precipitated by the presence of MgCl_2_, which prevented the oxidation of ^53^Cr(IIII). The data from Fig. S2 also show that the added ^50^Cr(VI) did not decrease during hot alkaline extraction procedure, as the peak area of ​​the 6 ng/mL ^50^Cr(VI) in the spiked sample (10-times diluted alkaline extract) (Fig. S2B) was the same as that of the 6 ng/mL isotopic ^50^Cr(VI) solution (Fig. S2A), prepared in 0.2% NaOH + 0.3% Na_2_CO_3_ + 0.04 M MgCl_2_. Data from the species interconversion experiment showed that no interconversion of Cr species occurred during the alkaline extraction procedure used, confirming the validity of the analytical data.

# RECOMMENDATIONS FOR COMPOSTING OF gRMW AND ITS FURTHER USE

Prior to composting, materials should be properly prepared: gRMW should be partially dried to a moisture level that allows the material to be further shredded (a few cm in diameter) and preferably mixed with biologically inert but physically stable structural material for aeration purposes (e.g., conifer bark chips). The mixture should then be moistened to the optimum water content as determined by the "fist test".

During the composting process, temperatures between 55 and 70 °C should be reached during two consecutive weeks to three consecutive days, respectively, to ensure sanitation (Regulation 2019/1009). Therefore, rapid composting in reactors is considered as a safe option. Other possible treatments (not tested in this study) would be thermal treatment (pasteurisation) using an external heat source at the beginning and subsequent composting at a lower technological and financial level.

At the end of the aerobic decomposition test, the moisture content of 100% gRMW was higher (65 ± 14%) than all other gRMW mixtures with compost. This could be due to the compactness of the material and the resulting inefficient aeration and evaporation of water. Therefore, we recommend mixing and turning the compost pile frequently when composting 100% gRMW to accelerate the drying of the material. Alternatively, the addition of homogeneous, inert substrate with low biodegradability, such as bark chips from coniferous trees, to 100% gRMW would promote loosening and drying of the material. The addition of compost to gRMW mixtures had some positive effects on the composting of the material - the resulting mixtures had lower moisture content (between 22 and 40%). However, in biological tests (germination, avoidance, and toxicity), 90% gRMW did not show favourable results.

Recommended application rates should be determined in further lysimeter test, but from our study we recommend the highest possible single application rate of up to 5% of the arable soil mass (i.e., up to 130 t gRMW/ha, assuming that 1 ha of a 20 cm deep soil layer weighs 2600 t). This high level of enrichment is recommended only for the production of so-called artificial soils, in which case gRMW would be an important physical and chemical improver of nutrient and structurally poor soils. A soil enriched with such a high amount of gRMW would increase the water holding capacity by up to 46 L/m^2^, which is sufficient for 9 days in a summer in temperate climate with intensive plant growth and high evapotranspiration (5 L/m^2^ per day).

For agronomic improvement of "normal" agricultural soils, we would recommend a much lower application rate, especially if applications are regular and repeated (e.g., every year or once every 5 years). In such a case, the application of 2 grams gRMW/L soil = 4 t/ha per year or better 20 t/ha every 5 years.

# LIST OF REFERENCES

Barly, S.H.Q., Okhrimenko, D. V., Solvang, M., Yue, Y., Stipp, S.L.S., 2019. Dissolution of Stone Wool Fibers with Phenol-urea-formaldehyde Binder in a Synthetic Lung Fluid. Chem. Res. Toxicol. 32, 2398–2410. <https://doi.org/10.1021/acs.chemrestox.9b00179>

BS EN 13650:2001: Soil improvers and growing media. Extraction of aqua regia soluble elements.

EU Regulation 1272/2008: Regulation (EC) No 1272/2008 of the European Parliament and of the Council of 16 December 2008 on classification, labelling and packaging of substances and mixtures, amending and repealing Directives 67/548/EEC and 1999/45/EC, and amending Regulation (EC) No 1907/2006. URL: <https://eur-lex.europa.eu/legal-content/EN/TXT/?uri=celex%3A32008R1272>.

EU Regulation 2019/1009: Regulation (EU) 2019/1009 of the European Parliament and of the Council of 5 June 2019 laying down rules on the making available on the market of EU fertilising products and amending Regulations (EC) No 1069/2009 and (EC) No 1107/2009 and repealing Regulation (EC) No 2003/2003. URL: <https://eur-lex.europa.eu/eli/reg/2019/1009/oj>.

Hoang, H.G., Thuy, B.T.P., Lin, C., Vo, D.V.N., Tran, H.T., Bahari, M.B., Le, V.G., Vu, C.T., 2022. The nitrogen cycle and mitigation strategies for nitrogen loss during organic waste composting: A review. Chemosphere 300, 134514. https://doi.org/10.1016/J.CHEMOSPHERE.2022.134514

ISO 10693:1995: Soil quality — Determination of carbonate content — Volumetric method.

ISO 10694:1996: Soil quality — Determination of organic and total carbon after dry combustion (elementary analysis).

ISO 11265:1996: Soil quality — Determination of the specific electrical conductivity.

ISO 15192:2010: Soil quality — Determination of chromium(VI) in solid material by alkaline digestion and ion chromatography with spectrophotometric detection.

ISO 16649-2:2001: Microbiology of food and animal feeding stuffs — Horizontal method for the enumeration of beta-glucuronidase-positive Escherichia coli — Part 2: Colony-count technique at 44 degrees C using 5-bromo-4-chloro-3-indolyl beta-D-glucuronide.

ISO 6579-1:2017: Microbiology of the food chain — Horizontal method for the detection, enumeration and serotyping of Salmonella — Part 1: Detection of Salmonella spp.

ISO 6887-1:2017: Microbiology of the food chain — Preparation of test samples, initial suspension and decimal dilutions for microbiological examination — Part 1: General rules for the preparation of the initial suspension and decimal dilutions.

Okhrimenko, D. V., Barly, S.H.Q., Jensen, M., Lakshtanov, L.Z., Johansson, D.B., Solvang, M., Yue, Y.Z., Stipp, S.L.S., 2022. Surface evolution of aluminosilicate glass fibers during dissolution: Influence of pH, solid-to-solution ratio and organic treatment. J. Colloid Interface Sci. 606, 1983–1997. https://doi.org/10.1016/j.jcis.2021.09.148
